# Effect of lactation stage and concurrent pregnancy on milk composition in the bottlenose dolphin

**DOI:** 10.1111/j.1469-7998.2007.00309.x

**Published:** 2007-10

**Authors:** K L West, O T Oftedal, J R Carpenter, B J Krames, M Campbell, J C Sweeney

**Affiliations:** 1 College of Natural Sciences, Hawaii Pacific University Kaneohe, HI, USA; 2 Nutrition Laboratory, National Zoological Park, Smithsonian Institution Washington, DC, USA; 3 Department of Human Nutrition, Food and Animal Sciences, University of Hawaii at Manoa, Agricultural Science 216 Honolulu, HI, USA; 4 318 S. East St., Culpeper, VA 22701, USA; 5 Dolphin Quest Bermuda, Bermuda Maritime Museum Sandys, Bermuda MA, USA; 6 Dolphin Quest/Quest Global Management San Diego, CA, USA

**Keywords:** bottlenose dolphin, *Tursiops*, milk composition, nutrition, lactation, Cetacea

## Abstract

Although many toothed whales (Cetacea: Odontoceti) lactate for 2–3 years or more, it is not known whether milk composition is affected by lactation stage in any odontocete species. We collected 64 pooled milk samples spanning 1–30 months postpartum from three captive bottlenose dolphins *Tursiops truncatus*. Milks were assayed for water, fat, crude protein (TN × 6.38) and sugar; gross energy was calculated. Ovulation and pregnancy were determined via monitoring of milk progesterone. Based on analysis of changes in milk composition for each individual dolphin, there were significant increases (*P*<0.05) in fat (in all three dolphins) and crude protein (in two of three), and a decrease (*P*<0.05) in water (in two of three) over the course of lactation, but the sugar content did not change. In all three animals, the energy content was positively correlated with month of lactation, but the percentage of energy provided by crude protein declined slightly but significantly (*P*<0.05). At mid-lactation (7–12 months postpartum, *n*=17), milk averaged 73.0±1.0% water, 12.8±1.0% fat, 8.9±0.5% crude protein, 1.0±0.1% sugar, 1.76±0.09 kcal g^−1^ (=7.25 kJ g^−1^) and 30.3±1.3% protein:energy per cent. This protein:energy per cent was surprisingly high compared with other cetaceans and in relation to the growth rates of calves. Milk progesterone indicated that dolphins ovulated and conceived between 413 and 673 days postpartum, following an increase in milk energy density. The significance of these observed compositional changes to calf nutrition will depend on the amounts of milk produced at different stages of lactation, and how milk composition and yield are influenced by sampling procedure, maternal diet and maternal condition, none of which are known.

## Introduction

Maternal care varies greatly in duration among mammals: in hooded seals, the mother weans her pup and departs to sea after 4 days or less (Bowen, Oftedal & Boness, 1985), while in some species of great apes, elephants and odontocetes (toothed whales and dolphins) lactation can last for more than 5 years, with the period of close association of the mother and young being even longer (Laws, 1969; Goodall, 1989; Mann *et al.*, 2000). This variation in the period of maternal dependence is presumably related to the need to learn social and cognitive skills, foraging techniques and predator-avoidance strategies (Hayssen, 1993). Some primates, proboscideans and odontocetes have complex social societies with highly context-specific interindividual interactions, and a great degree of social learning is essential to achieve social rank (Brodie, 1969; Horr, 1977; Goodall, 1989; Mann & Smuts, 1999; Sukumar, 2003). The difficulty involved in finding and catching food has been identified as a primary factor influencing the duration of lactation in cetaceans as offspring must be able to feed themselves before successful weaning (Whitehead & Mann, 2000).

However, there are nutritional consequences for both the mother and young of lactation patterns. Short lactations are typically intensive, with large volumes of nutrient-rich milk transferred to the young over a brief period of time (Oftedal, Bowen & Boness, 1996). Although highly demanding of the mother per unit time, the low overhead costs associated with fueling neonatal metabolism for just a brief period mean that maternal milk nutrients are converted to offspring body tissues with a very high efficiency, as much as 75–85% for fat and energy in some seals (Oftedal, Bowen & Boness, 1993; Lydersen & Kovacs, 1996; Oftedal *et al.*, 1996; Lydersen, Kovacs & Hammill, 1997; Lydersen & Kovacs, 1999). Thus, the total cost of lactation for the entire period is low. By contrast, in species with long nutritional dependence, mothers must cover the metabolic costs of their young for a prolonged period, resulting in much lower efficiency of offspring production, and much higher overall nutrient costs (Oftedal, 1985). In other words, the opportunity for extensive learning during prolonged lactation is achieved at a high nutritional and energetic cost.

These general comparisons are based on data for species with relatively short lactations (e.g. 1–4 weeks: true seals, some carnivores and some rodents) in comparison with species with lactations of moderate length (e.g. 3–6 months, such as bovids and cervids). Unfortunately, relatively little is known about nutrient transfer from the mother to young in species with extremely long lactations. In the wild, chimpanzees and orangutans lactate on average for 5 and 7.7 years, respectively (Knott, 2001; Kennedy, 2005), and African elephants lactate for an average of 4.4 years (Laws, 1969; Lee & Moss, 1986). Among odontocetes, bottlenose dolphin calves suckle for an average of 3–6 years in Shark Bay, Australia, with reports of mothers continuing to suckle calves for up to 8 years (Mann *et al.*, 2000). Based on the numbers of mothers and calves captured during drives, short-finned pilot whales are thought to nurse for 10–15 years (Kasuya & Marsh, 1984), while juvenile sperm whales may suckle for up to 12 years if reports of milk constituents among gastric contents are correct (Best, Canham & Macleod, 1984). Unfortunately, both of the latter reports are subject to alternative explanations, such as methodological error, adoptive suckling or milk stealing (Oftedal, 1997); it remains uncertain which mammal warrants the record for the longest lactation. In species with long lactations, it is also difficult to distinguish when lactation ceases to provide a significant nutrient supply, but rather serves simply as a social reinforcement (Oftedal, 1985).

Considering the crucial importance of nutrient supply to offspring physical, social and behavioral development, reliable information on the milk composition of toothed dolphin and whale milk is surprisingly scarce. Although reports of odontocete milk date back to the 1880s, samples have typically been collected from carcasses where lactation stage and calf health are unknown (Purdie, 1885; Eichelberger *et al.*, 1940; Pilson & Waller, 1970; Jenness & Odell, 1978; Ullrey *et al.*, 1984; Peddemors, de Muelenaere & Devchand, 1989). A recent review concluded that it was not possible from available data (19 publications) to determine whether milk varies in composition with lactation stage in any odontocete, as it does in baleen whales, pinnipeds and many other mammalian groups (Oftedal, 1997).

That this should remain unresolved even for bottlenose dolphins *Tursiops truncatus* is particularly surprising, as this species is prevalent in captivity and has been the subject of a substantial number of long-term behavioral and ecological studies in the wild (e.g. Wells, Boness & Rathbun, 1999; Connor *et al.*, 2000; Mann *et al.*, 2000; Wells, 2003). However, all published milk reports are based on only one or a few samples obtained opportunistically (Table 1). Although Ridgway *et al.* (1995) provide sequential data on the milk of several captive females induced to lactate by introduction of orphaned calves, it is not clear that this represents normal lactation. Otherwise, one can only conclude that bottlenose dolphin milk is highly variable in composition, ranging in reported lipid levels from 11 to 51% (Table 1). Such variable data are of little use in characterizing milk composition, whether to evaluate lactation costs, or as a model for the formulation of an artificial formula for rearing dolphin calves.

**Table 1:** Published compositional data on bottlenose dolphin *Tursiops truncatus* milk

	Source and number of samples	Collection method	Lactation stage	Water (%)	Fat (%)	Crude protein (%)	Sugar (%)
Eichelberger *et al.* (1940)	Captive (*n*=3)	Expression	Unknown	71.4	16.7	10.7	0.8
			Unknown	67.4	14.8	8.0	0.4
			‘Late’	75.5	10.8	12.2	0.6
Jenness & Sloan (1970)	Not stated (*n*=1)	Not stated	Not stated	58.3	33	6.8	1.1
Ackman, Eaton & Mitchell (1971)	Atlantic coast of Florida (*n*=1)	Postmortem	Probably late	–	19	–	–
Yablokov *et al.* (1974)	Not stated (*n*=1)	Not stated	Not stated	35.3	51.2	11.5	1.6
Pervaiz & Brew (1986*a*)	Captive (*n*=4)	Not stated	24–30 weeks	–	29.4	12.2	2.5
Peddemors *et al.* (1989)	Namibia (*n*=1)	Postmortem	2 years	72.8	13.2	12.1	–
Ridgway *et al.* (1995)^a^	Captive (*n*=2)	Expression	94–145 days	–	21.9	9.8	–
			169–180 days	–	20.4	9.8	–

a Samples obtained from animals induced to lactate, or 1–3 days after the death of a calf, were not included as they may not be normal.

This research aimed to (1) determine the changes in milk composition over the course of lactation in bottlenose dolphins, (2) to investigate the relationship between milk composition and reproductive state in lactating female dolphins, (3) to compare the milk of bottlenose dolphins with the milks of other cetaceans. We took advantage of the fact that bottlenose dolphins can be trained to present for voluntary milk collection and that progesterone concentrations can be determined from the milk whey fraction (West *et al.*, 2000). This method allows for non-invasive and daily biological sample collection to monitor the reproductive status of lactating female dolphins.

## Materials and methods

### Dolphins and sample collection

Three female bottlenose dolphins housed at Dolphin Quest facilities were studied: two at Dolphin Quest Hawaii located at the Hilton Waikoloa Village on the west coast of the island of Hawaii, 19°55′31 N, 155°53′19 W [Kona (Dolphin K) and Pele (Dolphin P)] and one at Dolphin Quest Bermuda located at the Fairmont Southampton Princess Resort on the south coast of the island of Bermuda, 32°15′01 N, 64°49′35 W [Bailey (Dolphin B)]. All three females and their suckling calves were kept together in lagoon settings and housed jointly with adult males, juvenile males and non-lactating female bottlenose dolphins. In Hawaii, the 150 by 200 ft. lagoon had a maximum depth of 15–18 ft., depending on tidal fluctuation, and contained *c*. 2 million gallons of natural sea water. In Bermuda, the dolphin facility comprised 2.5 acres of oceanic habitat with a maximum depth of 25 ft. All three pairs were healthy throughout the study period. The lactating females were fed similar diets composed of smelt, herring and mackerel. Food averaged *c*. 11.5 kg day^−1^ per lactating female throughout the study, except for the first 2–3 weeks after parturition, during which an additional 4.5 kg herring were added to the diet, totaling 16 kg day^−1^. Dolphins K and P were originally collected in the Gulf of Mexico and Dolphin B was born under the care of the Chicago Zoological Society at Hawks's Caye in the Florida Keys. Dolphins K and P gave birth in October 1997 and Dolphin B in May of 1998. Dolphin K was estimated at 13 years of age, Dolphin P at 12 years of age and Dolphin B was 9 years of age at the start of sampling. This was the first pregnancy for Dolphin B. Dolphin K had previously given birth to a surviving calf in 1994; although Dolphin P's first captive calf died shortly after birth in 1994, a second calf born in 1995 survived.

Milk samples were collected daily beginning *c*. 1 month after parturition. At Dolphin Quest, Hawaii, sampling continued until 27 months postpartum for Dolphin K and 29 months postpartum for Dolphin P. Approximately 700–800 individual samples were collected from Dolphins K and P and 400 from Dolphin B. Calves were weaned by trainers who separated the lactating mothers from their calves in preparation for upcoming births. Sampling at Dolphin Quest Bermuda was terminated at 15 months postpartum due to a severe hurricane that resulted in extensive damage to the enclosure and the permanent relocation of Dolphin B and her calf.

Dolphins were trained to present voluntarily for milk collection by animal handlers while remaining in the water. A human breast pump modified as a non-invasive milking device for dolphins (Kamolnick *et al.*, 1992) was used by animal trainers to collect an average of *c*. 4 mL milk at each sampling. An effort was made to avoid salt water contamination, including drying of the mammary area before the application of suction. We could not determine to what extent the glands were emptied, but given the small volumes, these samples probably represent a small fraction of the milk potentially available to suckling young. Milk samples were stored frozen in airtight containers at −15°C until thawed for pooling and analysis.

Milk samples were collected daily throughout the study period and samples from selected dates were analyzed for progesterone concentrations. In order to provide a sufficient volume of milk for gross compositional analyses, equal portions of the daily samples for each month were pooled. Milk samples were collected under approval of the University of Hawaii at Manoa Institutional Animal Care and Use Committee, protocol # 97-062-2.

### Laboratory analyses

The pregnancy status of the lactating bottlenose dolphins was monitored by measuring milk progesterone on approximately a weekly basis as described previously (West *et al.*, 2000). When progesterone concentrations above baseline were detected from the weekly sample, additional daily samples from the week before and following the progesterone spike were analyzed. Milk samples (1 mL) were centrifuged for 8 min at 2067 *g* to separate the milk into three visually distinct fractions: the uppermost fat layer, an aqueous whey fraction and precipitated solids. From the whey fraction, 100 *μ*L was directly analyzed for progesterone using a solid-phase radioimmunoassay kit (Diagnostic Products Corp., Los Angeles, CA, USA).

Milk fat was determined by the Babcock method (Horowitz *et al.*, 1975) in the Department of Human Nutrition, Food and Animal Sciences at the University of Hawaii at Manoa. Other proximate components were analyzed at the Nutrition Laboratory of the Smithsonian National Zoological Park, Washington, DC. Milk samples were warmed to 37°C and vortexed before subsampling. Dry matter composition of milk was determined by mass change of 100 *μ*L samples dried in a forced convection drying oven for 3 h at 100°C. The total nitrogen content of the milk was determined following combustion in an elemental gas analyzer (Perkin Elmer Model 2400 CHNS analyzer, Norwalk, CN, USA); combustion temperature and oxygen boosts were calibrated to yield results equivalent to the Kjeldahl method. Crude protein was calculated as 6.38 times the total nitrogen; no correction was made for non-protein nitrogen content. Total sugar concentrations were determined by the phenol–sulfuric acid calorimetric method (Marier & Boulet, 1959), with lactose monohydrate for the preparation of standards. All samples were run in duplicate or triplicate for each of the component analyses. Cow's milk samples were used as a control sample in each daily set of analyses and if the assayed values deviated from expected values by more than 10%, the entire daily sample set was re-run for that constituent. Gross energy (kcal g^−1^) for each sample was estimated indirectly from composition using a formula adapted from Perrin (1958): gross energy (kcal g^−1^)=9.11 ×% fat+5.86 ×% crude protein+3.95 ×% sugar (Oftedal, 1984). Crude protein and fat were also expressed as the percentage of gross energy.

For the purpose of this study, early, mid- and late-lactation stages were defined for bottlenose dolphins as 1–6 months, 7–12 months and greater than 12 months, respectively.

### Statistical analyses

Linear regression or correlational analyses were performed for each of the three individual dolphins on each proximate component, energy and crude protein energy per cent over the course of lactation (SIGMA STAT, SPSS Inc., Chicago, IL, USA). Data were tested for normality and homoskedasticity before regression analyses and in the case of fat, a reciprocal transformation was performed for each dolphin in order to meet these assumptions. Sugar and energy could not be consistently transformed among the three dolphins to meet these statistical assumptions. Spearman's rank order correlation was used to test for a correlation between the month of lactation and sugar, and the month of lactation and energy, for each dolphin. Spearman's rank order correlation was also used to investigate the relationship between milk fat and crude protein. Pearson product–moment correlations were used to determine the correlation between the average monthly water temperature and each milk proximate component, energy density and crude protein energy per cent.

## Results

There are no published reports on longitudinal change in milk composition from dolphins representing normal lactation (Table 1). We assayed a total of 69 pooled samples obtained from three dolphins that were sequentially sampled from the first month of lactation until 15, 27 and 29 months postpartum. The ranges of variation that we observed in milk water (59.5–81.1%), fat (6.8–25.2%), crude protein (5.1–12.7%) and sugar (0.4–2.5%) were large, but consistent with prior reports of bottlenose dolphin milk (Table 1), other than the apparently atypical high fat and low water values reported by Yablokov, Belkovich & Borisov (1974).

**Figure 1:**
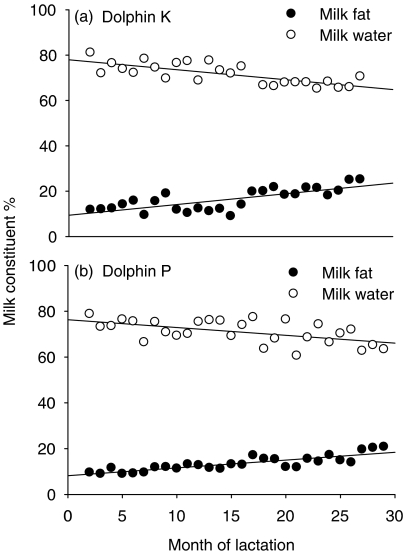
Changes in milk fat and milk water over the course of lactation in bottlenose dolphins *Tursiops truncatus*. Dolphin K (a) and Dolphin P (b).

**Figure 2:**
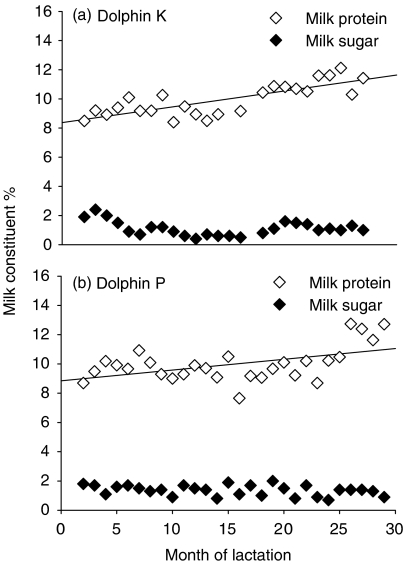
Changes in milk protein and milk sugar over the course of lactation in bottlenose dolphins *Tursiops truncatus*. Dolphin K (a) and Dolphin P (b).

Linear regression analyses for fat versus lactation stage (month of lactation) revealed significant linear relationships in all three of the dolphins. A significant increase (*P*<0.05) in fat was apparent in all three individuals and is illustrated for the two dolphins where sampling was not terminated early due to the permanent closure of the dolphin facility (Fig. 1a and b). The linear regression equations and *r*^2^ values are as follows for Dolphins K, P and B, respectively: 1.0/(milk fat)=[−0.00178 × (lactation month)]+0.0931, *r*^2^=0.435; 1.0/(milk fat)=[−0.00192 × (lactation month)]+0.108, *r*^2^=0.747; and 1.0/(milk fat)=[−0.00847 × (lactation month)]+0.163, *r*^2^=0.566. Linear regression analyses for water and protein versus lactation stage indicated significant linear relationships with month of lactation for Dolphins K and P, but not for Dolphin B. The percentage of water significantly decreased (*P*<0.05) over lactation for Dolphin K [water=(−0.439 × (month of lactation))+77.963; *r*^2^=0.553] and for Dolphin P [water=(−0.341 × (month of lactation))+76.322; *r*^2^=0.309] (Fig. 1a and b). Percentage of protein significantly increased (*P*<0.05) during the course of lactation for Dolphin K [protein=(0.019 × (month of lactation))+8.378; *r*^2^=6.14] and for Dolphin P [protein=(0.0736 × (month of lactation))+8.850; *r*^2^=0.253] (Fig. 2a and b). Sugar was the only constituent that showed no significant trend in any of the individual dolphins (Fig. 2a and b).

**Figure 3:**
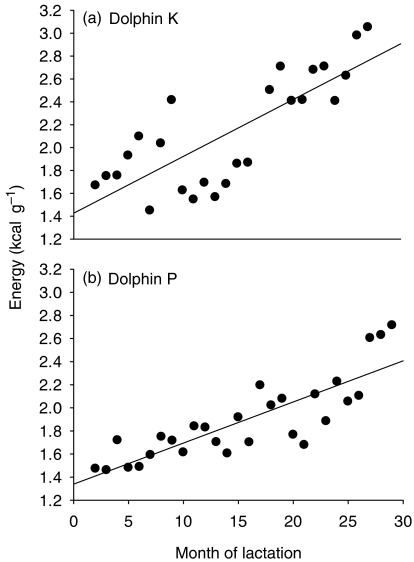
Changes in caloric energy density over the course of lactation in bottlenose dolphins *Tursiops truncatus*. Dolphin K (a) and Dolphin P (b).

**Figure 4:**
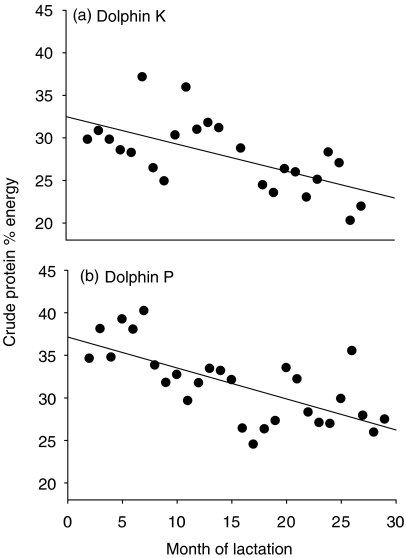
Changes in crude protein as a per cent of energy over the course of lactation in bottlenose dolphins *Tursiops truncatus*. Dolphin K (a) and Dolphin P (b).

Energy was positively correlated with month of lactation (*P*<0.05) in all three dolphins and is illustrated for Dolphins K and P (Fig. 3a and b). The correlation coefficient for energy and month of lactation was 0.710 for Dolphin K, 0.837 for Dolphin P and 0.745 for Dolphin B. Crude protein energy per cent significantly decreased (*P*<0.05) according to lactation stage in all three dolphins and is illustrated for Dolphins K and P (Fig. 4a and b). The linear regression equations and *r*^2^ values are as follows for Dolphins K, P and B, respectively: Crude protein energy =(−0.318 × month of lactation)+32.499, *r*^2^=0.386; crude protein energy =(−0.364 × month of lactation)+37.139, *r*^2^=0.471; crude protein energy =(−0.318 × month of lactation)+32.499, *r*^2^=0.577.

**Figure 5:**
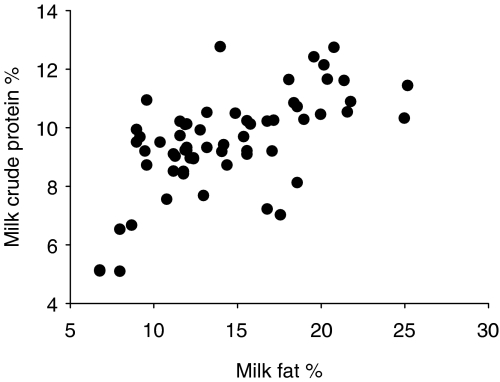
Relationship between milk crude protein and the milk fat of bottlenose dolphins *Tursiops truncatus*. Results are shown for data obtained from all three dolphins.

Milk crude protein was positively correlated with fat (*P*<0.05) and the ratio of fat:crude protein changed from *c*. 1.25 in early lactation to close to 2 in late lactation (Fig. 5). The correlation coefficient for milk crude protein and fat was 0.608.

The average monthly water temperature during the study period ranged between 17.8 and 28.3°C at Dolphin Quest Bermuda and between 23.2 and 27.3°C at Dolphin Quest Hawaii. Water temperature lows were recorded in both locations during the winter month of January and the highest temperatures between July and September. There was no significant correlation between water temperature and any of the following milk constituents: water, fat, crude protein, sugar, energy density or milk crude protein energy per cent.

**Figure 6:**
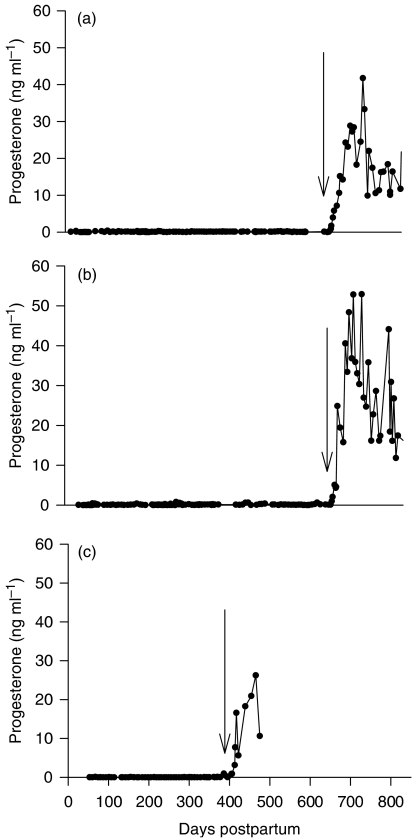
Postpartum progesterone concentrations of bottlenose dolphin *Tursiops truncatus* milk. The arrows indicate the first postpartum ovulation for each of the study dolphins. Dolphin K (a); Dolphin P (b); and Dolphin B (c).

**Figure 7:**
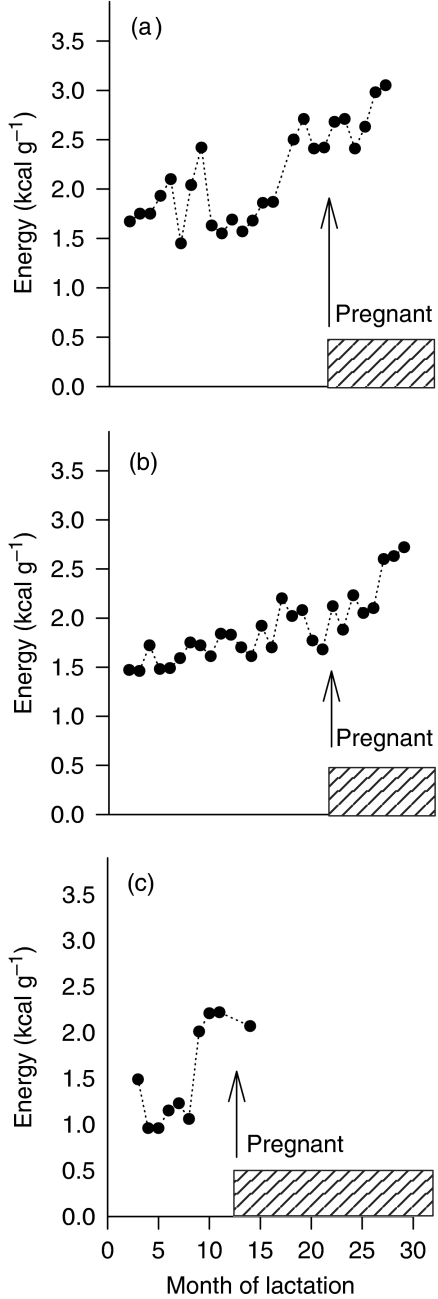
Energy density of bottlenose dolphin *Tursiops truncatus* milk in relation to the timing of the pregnancy. Dolphin K (a); Dolphin P (b); and Dolphin B (c).

The reproductive status in each dolphin was monitored by determining progesterone concentrations in the milk samples. Progesterone concentrations did not fluctuate above a baseline value of 1 ng mL^−1^ (West *et al.*, 2000) from the start of sampling until 413 days postpartum for Dolphin B and 673 days postpartum for Dolphins K and P. The first increase above baseline progesterone values signified the first detectable postpartum ovulation in each dolphin and in all three cases, once progesterone levels increased, the values remained at high but variable levels for the rest of the study period (Fig. 6a–c). Progesterone concentrations reached a high of 26.3 ng mL^−1^ in Dolphin B, 41.2 n g mL^−1^ in Dolphin K and 48.4 ng mL^−1^ in Dolphin P. Pregnancy in the three study animals was confirmed by ultrasound evaluation several months after conception. In all individuals, the first detectable postpartum ovulation occurred at a point in lactation with a relatively high milk fat and crude protein content, which is reflected in the total energy density of the milk. The relationship of milk energy density with the timing of pregnancy is illustrated for the three individual study dolphins in Fig. 7a–c. The energy density ranged between 1 and 1.5 kcal g^−1^ during early lactation, between 1.7 and 2.7 kcal g^−1^ during early pregnancy and between 2.5 and 3.0 kcal g^−1^ at 30 months of lactation (6–8 months into pregnancy) for the two dolphins in Hawaii.

## Discussion

Maternal investment can be measured if the quality of milk is determined and the amount transferred to offspring is quantified. Milk intake has been measured in a number of terrestrial mammals and in pinnipeds that nurse their young on land using isotopic dilution techniques (Holleman, White & Luick, 1975; Oftedal, 1984; Oftedal *et al.*, 1987; Dove, 1988; Lydersen & Kovacs, 1999). However, cetaceans spend the entire nursing period immersed in water, and this factor, combined with their large size and the inherent difficulties with isotope administration and sequential blood sample collection, has precluded the quantification of milk intake in any cetacean. The present study addresses solely the quality of milk produced but is the first definitive report on change in the milk composition of a cetacean based on longitudinal samples from females with known-age calves.

### Importance of lactation stage

Lactation is a defining characteristic of mammalian reproduction that involves substantial investment of energy and nutrients in the young and thus has a major impact on maternal nutrient requirements (Oftedal, 1984, 1985). Given the potential importance of lactation to the life history, foraging and reproductive strategies of mammals, it is surprising that cetacean lactation has received relatively little attention (Hayssen, 1993; Oftedal, 1997). The comparative evaluation of cetacean milks has been hindered by the incomplete and uneven nature of available data (Oftedal, 1997). Although the first reports on cetacean milks date to the late 19th century (Purdie, 1885; Frankland & Hambly, 1890), for the next hundred years most milk samples were collected from animals killed during whaling operations or during local harvests, or from stranded animals, and unless an accompanying calf could be captured and aged, the stage of lactation was unknown. This lack of specified lactation stage precluded inclusion of cetaceans in reviews of mammalian milk that focused on mid-lactation (e.g. Oftedal, 1984). However, in seasonal breeders with known migratory patterns during the reproductive cycle, such as many mysticete whales, lactation stage may be roughly estimated from the date and site of capture, and can occasionally be substantiated from the size of accompanying calves (e.g. Chittleborough, 1958; Best, 1982; Best *et al.*, 1984). From such reports, it appears that milk composition changes with lactation stage at least in such mysticetes as minke whales *Balaeonoptera acutorostrata*, fin whales *Balaeonoptera physalus* and humpback whales *Megaptera novaeangliae* (Oftedal, 1997). By contrast, Oftedal (1997) was unable to reliably assign lactation stage to most milk samples from odontocetes, and from limited data for 15 species concluded that ‘it is not possible to determine if milk composition changes over the course of lactation among odontocetes.’

In bottlenose dolphins, milk water was found to decline as milk fat increased over the first 1–2.5 years of lactation (Fig. 1a and b). This is consistent with similar apparent trends observed during the *c*. 5 month lactation in minke whales and the 6–8 month lactation in fin whales (Oftedal, 1997). In humpback whales, it appears that water declines and fat increases only over the first 5–7 months of lactation, after which water increases and fat declines until weaning at 10–12 months (Chittleborough, 1958; Symons & Weston, 1958; Oftedal, 1997).

### Variation in fat content

The peak fat level reached by bottlenose dolphin milk in late lactation (17% fat) is lower than the 20–47% fat reported for other cetaceans at mid- to late lactation (Table 2). Given the great variability observed in fat content of bottlenose dolphin milks (Fig. 1) as well as in the milks of humpback, minke, fin and blue whales *Balaeonoptera musculus* (Oftedal, 1997), it is inadvisable to attempt to characterize milk composition for any cetacean from just one or a few samples. Table 2 is therefore restricted to species in which at least 3–5 samples have been assayed at mid–late lactation. The two odontocetes, spotted dolphins *Stenella attenuata* and killer whales *Orcinus orca*, are similar to bottlenose dolphins in having moderate fat levels (24–25% on average), whereas at least in late lactation the mysticetes (humpback whale, minke whale, fin whale, blue whale) produce milks considerably higher in fat (30–47%) (Table 2). Whether this is a consistent difference between the two suborders is not certain. Based on single samples, a few odontocetes have been reported to produce milk containing >30% fat, including common dolphins *Delphinus delphis* off South Africa (Peddemors *et al.*, 1989) and long-finned pilot whales *Globicephala melas* and harbor porpoises *Phocoena phocoena* near Scotland (Purdie, 1885; Frankland & Hambly, 1890), but these preliminary findings require confirmation. The highest fat value we measured for the bottlenose dolphin was 25.2%, although Pervaiz & Brew (1986*a*) and Jenness & Sloan (1970) reported samples containing 29 and 33% fat, respectively (Table 1). We suspect the 51.2% fat cited for *T. truncatus* milk in a poorly referenced compilation by Yablokov *et al.* (1974) to be erroneous.

**Table 2:** Cetacean milk composition at early, mid- and late lactation

Species	Lactation stage and sampling method^a^	*n*^b^	Water (%)	Fat (%)	Crude protein^c^ (%)	Ash (%)	Sugar^d^ (%)	Gross energy^e^ (kcal g^−1^)	Protein:energy (%)	Source
Bottlenose dolphin *Tursiops truncatus*	1–6 months, man expr	14	75.3 ± 0.9	10.5 ± 0.7	8.5 ± 0.5	–	1.4n ± 0.5	1.53 ± 0.27	32.6 ± 1.0	This study
	7–12 months, man expr	17	73.0 ± 1.0	12.8 ± 1.0	8.9 ± 0.5	–	1.0n ± 0.1	1.76 ± 0.09	30.3 ± 1.3	This study
	13–29 months, man expr	32	70.0 ± 0.9	16.6 ± 0.7	10.5 ± 0.3	–	1.1n ± 0.1	2.20 ± 0.07	28.4 ± 1.1	This study
Spotted dolphin *Stenella attenuata*	Unknown, post-m.	8	–	25.3 ± 1.9	9.2 ± 0.3	–	1.1r ± 0.1	2.89 ± 0.18	19.2 ± 1.5	Pilson & Waller (1970)
Sperm whale *Physeter macrocephalus*	∼1–3 months, post-m.	5	64.3 ± 2.1	23.6 ± 1.9	10.2u ± 0.4	0.8 ± 0.04	*1.2d*±*0.1*	2.79 ± 0.18	21.7 ± 1.2	Best *et al.* (1984)
	∼3–5 months, post-m.	7	63.8 ± 1.7	25.7 ± 1.6	8.5u ± 0.3	0.6 ± 0.04	*1.4d*±*0.4*	2.90 ± 0.15	17.5 ± 0.8	Best *et al.* (1984)
Humpback whale *Megaptera novaeangliae*	∼6–7 months, post-m.	3	40.1 ± 0.6	47.3 ± 1.2	9.10 ± 0.3	2.1 ± 0.19	0.66r ± 0.2	4.87 ± 0.12	10.9 ± 0.3	Symons & Weston (1958)
	∼10 months, post-m.	5	49.8 ± 2.8	35.1 ± 2.8	12.3 ± 0.5	1.5 ± 0.04	*1.2d*±*0.3*	3.97 ± 0.26	18.5 ± 1.4	Chittleborough (1958)
Minke whale *Balaeonoptera acutorostrata*	∼2–4 months, post-m.	7	60.8 ± 4.7	20.3 ± 4.4	14.6u ± 0.8	1.7 ± 0.23	*2.5d*±*0.7*	2.81 ± 0.40	35.2 ± 5.8	Best (1982)
	∼4–5 months, post-m.	16	51.9 ± 3.2	30.2 ± 3.2	13.6u ± 0.7	1.7 ± 0.11	*2.7d*±*0.3*	3.65 ± 0.29	23.7 ± 2.3	Best (1982)
Fin whale *Balaeonoptera physalus*	∼4–7 months, post-m.	9	51.5 ± 2.4	34.9 ± 2.7	11.4 ± 0.5	1.2 ± 0.17	1.4r ± 0.4	3.91 ± 0.23	17.7 ± 1.6	Takata (1922), White (1953), Ohta *et al.* (1955), Lauer & Baker (1969)
Blue whale *Balaeonoptera musculus*	∼5–7 months, post-m.	5	48.1 ± 3.8	36.7 ± 4.9	12.0 ± 0.5	1.4 ± 0.07	*1.7d*±*1.0*	4.12 ± 0.40	18.0 ± 2.4	Backhaus (1904), White (1953), Gregory *et al.* (1955)

a Exact lactation stage was known only for captive bottlenose dolphins; in other species, lactation stage was estimated by comparison with peak calving dates (if possible) and are approximate (see Oftedal, 1997). Methods of collection: Man expr, manual expression during voluntary restraint; post-m., postmortem collection from nipple after the animal drowned in a net (spotted dolphin) or from nipple or excised mammary gland after the animal was harpooned and hauled onto a flensing deck or platform (whales).

b Numbers of samples for which fat, protein and sugar were assayed or (for sugar by difference) calculated. Any samples reported to be contaminated or derived from involuting mammary glands were excluded.

c Protein equals crude protein, that is, total nitrogen × 6.38 unless otherwise specified; u, unknown (analytical method not reported).

d Values in italics and followed by *d* were calculated by difference, that is, 100–(water%+fat%+protein%+ash%) and other values were obtained by the following methods: r, reducing sugar method; *n*, non-specific phenol–sulfuric acid method.

e Gross energy calculated per Oftedal (1984) as (fat%× 9.11+protein%× 5.86+sugar%× 3.95)/100. No correction was made for non-protein nitrogen.

The great intra-animal variability in fat content (Fig. 1a and b) is not solely due to lactation stage, but we can only guess at other factors that may come into play. Salt water contamination during voluntary sample collection could present a confounding factor for some samples, despite efforts to avoid it. Water contamination would depress all constituents, not just fat, and the fact that our assays were based on pooled milks derived from a large number of individual samples should minimize the effect of any particular contamination event during collection. It is possible that milk fat could change with the degree of mammary emptying, such that samples collected before and after a suckling event might differ. For example, in many domestic or dairy animals (including camels, cattle, sheep and pigs) and in humans, milk fat increases over the course of milk expression so that the first sample drawn from full mammary glands may contain but one-third or less of the fat in samples of ‘residual milk’ obtained from glands that have been emptied by suckling or machine milking (Oftedal, 1984; Atwood & Hartmann, 1992; Tesorero *et al.*, 2001). As our small individual samples were collected from trained dolphins during voluntary procedures, they would have included samples obtained both before and after calf suckling, which might increase variability in measured fat levels. However, the fact that many individual samples were pooled should have averaged out much of this potential variability.

Dolphin calves suckle frequently (Cockcroft & Ross, 1990; Peddemors, Fothergill & Cockcroft, 1992; Jacobsen, Mayntz & Amundin, 2003), so that it is unlikely that dolphin mammary glands become highly engorged. Nonetheless, it would be valuable to examine variability in samples in which a greater proportion of mammary contents is removed. In terrestrial mammals, exogenous oxytocin is often used to induce milk letdown and thereby express a greater fraction of mammary contents during milking. It would be valuable to compare sequential milk samples obtained this way to determine whether milk fat levels increase during milk expression in dolphins or other cetaceans. If they do, our small voluntary samples may underestimate average fat content of mammary secretion. By contrast, those pinnipeds that have been studied do not demonstrate a rise in fat among sequential milk samples (Oftedal *et al.*, 1996). In such a case, small samples would not produce much sampling bias or variability in results.

Another potential source of variation in milk composition is associated with maternal condition and diet. However, all of our females were well nourished and in good condition, and the amounts fed were stable. Differences in diet or condition could contribute to apparent differences among species, or between captive and wild animals, but do not explain the within-animal variability we observed.

### Protein and postnatal growth

Most of the nitrogen in bottlenose dolphin milk is true protein, although non-protein nitrogen has been measured as 0.12–0.13% (Eichelberger *et al.*, 1940). Thus, crude protein (total nitrogen × 6.38) overestimates true protein (protein nitrogen × 6.38) by about 0.8 percentage points. Previous studies have indicated that caseins comprise about 57%, and whey proteins (including two *β*-lactoglobulins and *α*-lactalbumin) about 43% of milk protein (Jenness & Sloan, 1970; Pervaiz & Brew, 1986*a*,*b*). In bottlenose dolphin milk, crude protein varied over a smaller range than fat, but the two were positively correlated (Fig. 5). As milk energy content was also changing over lactation (Fig. 3a and b), even though crude protein increased, the proportion of energy supplied by protein declined gradually, from about 35% at the beginning of lactation to about 28% at 2.5 years (Fig. 4a and b). This range is higher than the values calculated for most other cetaceans (Table 2). With the exception of minke whales in early lactation (35%) and humpback whales in mid-lactation (11%), the average protein:energy per cent fell within the rather narrow range of 18–24% in other odontocetes and mysticetes. Note that these estimates are based on energy calculated from sugar content that may include error. However, as the estimated sugar energy content (sugar × 3.95) only accounts for 0.03–0.11 kcal g^−1^, or about 0.5–3.5% of the total energy, depending on the species, even a large error in the estimated sugar content will not have much impact on either the calculated total energy content or the percentage of energy provided by protein.

Although high relative to other cetaceans, the protein levels in bottlenose dolphin milk are comparable, on an energy basis, to what is found in some orders of terrestrial mammals. Rodents, lagomorphs and terrestrial carnivores (other than denned bears) produce mid-lactation milks containing 22–46, 29–39 and 27–50% of energy as protein (Oftedal, 1984; Gittleman & Oftedal, 1987; Derrickson, Jerrard & Oftedal, 1996). The high milk protein in these taxa may be necessary to support rapid postnatal growth, but this explanation does not appear to hold for dolphins. In fact, bottlenose dolphins and other odontocetes have both low rates of postnatal growth and prolonged periods of dependency, as do primates (Oftedal, 1997). Based on the postnatal growth rate, one might expect bottlenose dolphins to produce milks with a protein:energy per cent of 22% or less, as do most primates (Power, Oftedal & Tardif, 2002).

The reason for the relatively high protein:energy per cent in dolphin milk, both in comparison with wild cetaceans and to other mammals that grow slowly, is not clear. A difference in milk energy content could occur between captive and wild cetaceans, but as captive cetaceans are more apt to be in good condition and on a high plane of nutrition, the predicted difference would be lower fat and energy, and thus higher protein:energy ratios, in the wild. High-protein milk could provide a protein supplement during the gradual weaning transition that occurs after 6–12 months (Mann & Sargeant, 2003), but as most dolphin prey (such as fish and squid) are likely high in protein this seems unlikely. It is possible that dolphin calves have higher protein requirements than would be predicted from comparative growth, or that lactating dolphins simply secrete protein in excess of calf requirements. Further research is needed on calf food habits and nutrient requirements to resolve these issues.

### Sugar in cetacean milks

The sugar content of bottlenose dolphin milk, as measured by the non-specific phenol–sulfuric acid method, ranged from 0.4 to 2.4%, but did not change significantly over the course of lactation (Fig. 2a and b). The values for early, mid- and late-lactation averaged 1.0–1.4% (Table 2). The phenol–sulfuric acid method measures monosaccharides, disaccharides and oligosaccharides, although it may omit small quantities of amino sugars (Oftedal & Iverson, 1995).

The sugars in dolphin milks are more complex than originally thought. According to Pervaiz & Brew (1986*a*), lactose is the predominant sugar in dolphin milk at mid-lactation, but Oftedal (1997), citing Newburg, D. S., (unpublished), indicated that oligosaccarides are also present. Uemura *et al.* (2005) recently determined that dolphin milk obtained 2 days postpartum contained 0.9 g oligosaccharides, 1.6 g lactose and 0.3 g monosaccharides per 100 mL. The primary free oligosaccharides were a novel monosialyl–tetrasaccharide [GalNAc(*β*1–4)[Neu5Ac(*α*2–30]Gal(*β*1–4)Glc], 3′-sialyllactose, 6′-sialyllactose and globotriose [Gal(*α* 1–4) Gal(*β*1–4)Glc]. Neither the free monosialyl–tetrasaccharide nor globotriose had previously been identified in mammalian milks (Uemura *et al.*, 2005).

The presence of oligosaccharides in cetacean milks (Urashima *et al.*, 2002; Uemura *et al.*, 2005) undermines the accuracy of reducing sugar methods that have been routinely used to assay sugar in cetacean milks for many years (Oftedal, 1997; Table 2). When applied to milk, reducing sugar methods assume that measured sugars have a reducing power comparable to lactose, but this assumption is incorrect for oligosaccharides. Thus, the three sugar estimates in Table 2 obtained by reducing sugar methods likely underestimate the total sugar in these species. However, such data are still preferable to estimating sugar by difference, that is, 100−(water%+fat%+crude protein%), as this approach combines the errors of all other assays. Most of the sugar data in Table 2 were calculated by difference and thus should be considered suspect. We recommend that future investigations of cetacean milks use a non-specific sugar method such as the phenol–sulfuric acid method. Fortunately, the sugar contents of dolphin milks are low and therefore the impact of analytic error on overall energetic calculations remains minimal.

### Lactation and concurrent pregnancy

In most mammals, the suckling stimulus acts to inhibit ovulation in lactating mothers (McNeilly, 1997). However, it is not uncommon for dolphins to ovulate while lactating as evidenced by the concurrent pregnancy of all three of our study animals and by the simultaneous lactation and pregnancy previously described for captive bottlenose dolphins and wild-striped dolphins *Stenella coeruleoalba* (Yoshioka, Aida & Hanyu, 1989; West *et al.*, 2000). In the wild, there are reports of bottlenose dolphins continuing to produce milk for 6–8 years postpartum (Mann, 1999; Wells, 1999; Mann *et al.*, 2000; Mann & Sargeant, 2003). It may be the degree of suckling activitiy, not suckling per se, that impacts the duration of lactational infertility in dolphins. Suckling is believed to interfere with the hypothalamic release of the GnRH, which suppresses LH release in other mammals (McNeilly, 1997; Montiel & Ahuja, 2005). To date, studies of suckling frequency in cetaceans have focused on the first few weeks to months postpartum (Cockcroft & Ross, 1990; Clark & Odell, 2000; Jacobsen *et al.*, 2003). Peddemors *et al.* (1992) investigated long-term (+1 year) suckling frequency in a single bottlenose dolphin calf but the reproductive status of the mother was not evaluated. The relationship between GnRH and LH concentrations of the mother and the suckling frequency of the calf is yet to be determined in any cetacean.

The timing of the first postpartum ovulation has been identified as a primary factor limiting the reproductive efficiency of cattle (Montiel & Ahuja, 2005) and undoubtedly has an impact on the reproductive interval of dolphins. In addition to suckling frequency, nutrition of the mother influences the resumption of ovarian activity in both cows and sows (Butler & Smith, 1989; Montiel & Ahuja, 2005). Dietary intake and mobilization of body stores has been related to the timing of the first postpartum ovulation in cattle (Butler & Smith, 1989), indicating that a negative energy balance resulting from the high cost of lactation suppresses ovulation. The first postpartum ovulation occurred in two of our dolphins at 673 days postpartum and at 413 days postpartum in the third dolphin (Fig. 6). Interestingly, ovulation followed a gradual increase in milk fat and milk protein in each of the lactating females; milk fat and protein, and therefore milk energy, continued to increase as simultaneous lactation and pregnancy progressed (Fig. 7a–c). The production of milk with both high fat and protein content may reflect well-fed dolphins having substantial body stores, as reported in other taxa (Rasmussen, 1992; Lalman *et al.*, 2000; Tardif *et al.*, 2001). We do not know whether these compositional trends would hold in wild animals under adverse dietary conditions, or for captive animals on more restrictive diets. Further research is needed on milk composition in relation to nutrient intakes, body condition and reproductive status in order to understand the interplay between dolphin reproduction and nutrition.
